# Control of hyperphosphatemia and maintenance of calcemia in CKD

**DOI:** 10.1590/2175-8239-JBN-2021-S105

**Published:** 2021-12-03

**Authors:** Aluizio Barbosa Carvalho, Fabiana Baggio Nerbass, Lilian Cuppari

**Affiliations:** 1 Universidade Federal de São Paulo, São Paulo, SP, Brazil.; 2 Fundação Pró-Rim, Joinville, SC, Brazil.

## 1. Control of serum phosphorus and calcium levels in CKD

1.1 In adults with CKD G3a-5D, receiving treatment to reduce phosphorus overload, decisions should be based on persistent or progressively elevated serum phosphorus levels (Opinion).

1.2 In adults with CKD G3a-5D, phosphorus levels should be maintained within the normal range (Evidence).

1.3 In adults with CKD G3a-5D, with hypercalcemia or presence of vascular calcification, calcium-containing phosphate binders should be avoided (Evidence).

1.4 In adults with CKD G5D, calcium concentration in the dialysate should preferably be 3.0 mEq/L (Opinion).

1.5 To adults with CKD G3a-5D undergoing hyperphosphatemia treatment, the dose of calcium-based phosphate binders should be restricted (Opinion).

1.6 For adults with CKD G3a-5D, the use of aluminum-containing phosphate binders should be avoided, and in those on dialysis, the aluminum concentration in the dialysate should be continuously monitored (Evidence).

1.7 For adults with CKD G5D, with persistent hyperphosphatemia, measures to increase phosphorus removal by dialysis should be implemented (Opinion).

## 2. Assessment of phosphorus intake and dietary guidance

2.1 Assessment and guidance regarding phosphorus intake should be performed by a nutritionist (Opinion). 

2.2 For adults with CKD G3a-5D, it is recommended to assess and, if necessary, adjust phosphorus intake to maintain phosphatemia within the normal range (Evidence). It is advisable to consider the food source of phosphorus (animal, vegetal, phosphorus additives) (Opinion).

2.3 For adults with CKD G5D, the adjustment in phosphorus intake should consider the recommended protein intake of 1.0 to 1.2 g/kg body weight/day (Opinion).

2.4 For adults with CKD G3a-5, adjustment in phosphorus intake should consider the recommended protein intake of 0.6 to 0.8 g/kg body weight/day (Opinion).

2.5 Adults with CKD G3a-5D should take phosphate binders in meals/ snacks containing significant amount of phosphorus (Opinion). 

## RATIONAL

Hyperphosphatemia in CKD results from some main factors: reduced phosphate (P) clearance (kidney and by dialysis methods), bone turnover status (high or low), use of vitamin D analogues, inadequate use of binders, and excessive phosphorus intake. P retention and/or hyperphosphatemia are among the factors contributing to the development of secondary hyperparathyroidism (SHP) in CKD patients. Hyperphosphatemia is also associated with morbidity and mortality in these patients, mainly related to cardiovascular events^1^ ^,^ ^2^. The mechanisms by which P retention increases the risk of cardiovascular events and mortality are not yet fully clarified^3^ ^,^ ^4^. These mechanisms involve the phenotypic transformation of smooth muscle cells of the medial layer of arterial vessels, induced by P or indirectly by the effects of hyperphosphatemia on PTH, triggering SHP and vascular calcification^5^ ^,^ ^6^. The rational for preventing P retention or treating established hyperphosphatemia lies in its known role in the development of SHP. In addition, other unproven benefits would be decreased risk of vascular and soft tissue calcification, prevention of cardiovascular events and CKD progression. Available evidence supports that serum P values, lower or higher than the normal range, are associated with worse outcomes, including death^2^ ^,^ ^7^. However, recommended P levels associated with a better prognosis are difficult to determine. In CKD stages 2-4, studies assessing this aspect are scarce. It is known that serum P levels above 3.5 mg/dL, in pre-dialysis patients, are associated with increased mortality.^8^ In CKD stage 5D, findings from observational studies indicate different values associated with risk of cardiovascular complications or death. However, an analysis of a cohort of 40,000 prevalent HD patients has demonstrated that the risk of death increases when plasma P is above 5.0 mg/dL^2^. Thus, evidence suggests that serum P levels within the normal range are associated with better outcomes. However, there is still a need for intervention studies that might more accurately identify optimal P levels for CKD patients. Studies show that the serum P concentration remains within the normal range until the GFR declines at 20 to 30 mL/min.^8^

### Dietary assessment and guidance

 The treatment of hyperphosphatemia involves a multidisciplinary approach, since its cause is multifactorial and it includes not only dietary aspects, but also those related to inefficient phosphorus removal by dialysis, use of vitamin D analogues, characteristics of mineral and bone metabolism disorders, and inappropriate use of phosphate binders^9^.

It is important to highlight that, before any dietary intervention, it is of paramount importance to know the patient's food intake. Although methods for assessing food intake have limitations, especially from a quantitative point of view, using multiple 24-hour dietary recall, multiple days of food diary entry, food frequency questionnaire, or even a detailed survey of usual intake allows the nutritionist, with good knowledge of foods that are a source of phosphorus, to identify if there is and what is the influence of food on hyperphosphatemia. This is fundamental so that mistaken prejudgment is not made and restrictions are not excessive, situations that could lead to low adherence and worsening of the nutritional status and of the diet quality, besides hindering the establishment and/or maintenance of the bond with the patient. In addition, this assessment provides support for investigating other causes, especially when there is no clear correlation between phosphorus intake and hyperphosphatemia. Traditionally, it has been recommended that phosphorus intake should be maintained between 800-1000 mg/day, in order to maintain phosphatemia within normal ranges^10^ ^,^ ^11^. However, the effectiveness of this recommendation has not been established.

Phosphorus is distributed in a wide variety of foods in organic and inorganic forms. Organic phosphorus is found naturally, mostly in foods that are a source of protein, either of plant or of animal origin. However, phosphorus from animal source foods is absorbed more efficiently in the gastrointestinal tract (GIT) than that from plant source foods (approximately > 70% vs. < 40%, respectively)^12^ ^,^ ^13^. In vegetables, most of the phosphorus is complexed to phytate (a carbohydrate not digestible by the GIT enzymes), making its absorption difficult. Inorganic phosphorus, on the other hand, which may be absorbed by the GIT up to 100%, is found in chemical additives used in processed and ultra-processed foods. Consumption of these foods has increased in recent decades, which may contribute to excessive phosphorus intake in the general population and to phosphate overload in CKD^14^. Another potential source of phosphorus, generally neglected, comes from medicines and food supplements, especially those containing a wide range of vitamins and minerals^15^. 

As shown in Table 1, the main sources of phosphorus are also those high-protein foods, such as meat, eggs, and dairy products (except butter). In the non-dialytic phase of CKD, control of protein intake (0.6 to 0.8 g/kg/day) already limits the amount of phosphorus in the diet, but it is important to remember that this control is not always easy to achieve and that processed/ultra-processed foods may also contribute significantly to the total amount of phosphorus ingested. 

Some controlled clinical trials assessing the effect of a phosphorus-restricted diet, associated or not with a low-protein diet in the non-dialytic phase of CKD, have observed a reduction in serum and urinary phosphorus after the intervention^16^. In studies where protein intake was even more tightly controlled, such as in the very low-protein diet supplemented with ketoacids, serum phosphorus also decreased significantly in most of them^17^ ^-^ ^19^.

**Table 1.** Main foods that are a source of protein and phosphorus

Food	Quantity (g)	Home Measure	P (mg)	Protein (g)	P/protein ratio (mg/g)
Chicken meat	80	1 medium breast fillet	150	23	6.5
Pork	80	1 medium steak	147	21.2	6.9
Beef	85	1 medium steak	209	26	8
Hake	84	1 medium fillet	241	20.6	11.7
Whole egg	50	1 unit	90	6	15
Egg white	30	1 unit	4,3	3.3	1.3
Beef liver	85	1 medium steak	404	22.7	17.8
Sardines	34	1 unit	170	8.4	20.2
Ham	48	2 medium slices	136	14	9.7
Prato cheese	30	2 thin slices	153	7.5	20.4
Yogurt	120	1 small cup	159	6.3	25.2
Milk	150	1 cup (250 ml)	140	4.9	28.6
Cooked soy	54	5 tablespoons	130	9	14.5
Cooked beans	154	1 medium ladle	133	6.9	19.3
Peanut	50	1 small package	253	13	19.9
Chocolate	40	1 small bar	92	3	30.7

Adjustments in P intake should be made carefully so as not to cause excessive reduction in serum concentration, since hypophosphatemia may indicate insufficient protein intake, besides being associated with a higher risk of morbidity and mortality^20^.

In CKD stage 5D (dialysis), assessment of phosphorus intake as well as other factors contributing to hyperphosphatemia is necessary when serum phosphorus is persistently elevated, since dialysis methods are relatively inefficient in removing it. Adjustment in phosphorus intake should be made with caution, so as not to compromise protein intake, which should be maintained between 1.0 to 1.2 g/kg/day at this stage of CKD. High-protein foods are naturally high in P and contribute with a significant part of ingested P. One way to provide the required amount of protein, with the lowest possible P content, is to select foods with the lowest P/protein ratio, as shown in Table 1. A study of hemodialysis patients has demonstrated for the first time that the risk of death was 2.37 times greater in the highest tertile of P intake compared to the lowest tertile. The risk was also greater in the group of patients with a dietary P/protein ratio above 16 mg/g.^21^ In addition, it is important to reduce processed foods that contain P-based additives (phosphoric acid, polyphosphates and pyrophosphates), such as semi-prepared foods, so-called fast foods, sausages, processed cheeses, instant products, cookies, breakfast cereals and cola-based soft drinks. A national study found very high phosphorus concentrations in domestic industrialized products commonly consumed by dialysis patients in the Southeast region^22^. There is evidence that restricting foods containing P additives promotes a reduction in phosphatemia in HD patients^23^. A randomized controlled clinical trial, also performed in Brazil, showed an important and significant reduction of phosphatemia in patients who received nutritional orientation to avoid the consumption of processed foods in preference to fresh foods, with the maintenance of protein intake^24^. Individualized dietary guidance by nutritionists, associated with nutrition education programs, is fundamental to improve patient adherence^25^. A recent meta-analysis, which included only randomized controlled trials that used strategies to improve adherence to hyperphosphatemia treatment, found a significant reduction in phosphorus concentrations in patients submitted to the interventions^26^. Chart 1 shows the four general orientations recommended by the National Kidney Foundation guidelines^27^.

**Chart 1.** Recommendations to patients for phosphatemia control

Choose preferably fresh foods, with lower bioavailability of phosphate. When consuming processed foods, choose those that do not have phosphate additives in their composition. Prefer protein sources with lower mg of phosphorus per g of protein ratio. Recommend preparing meals at home, preferring, whenever necessary, moist heat cooking methods such as boiling, always discarding the cooking water.

Source: National Kidney Foundation Guidelines.

A tool that might be used to help in nutritional guidance is the *Dietary Guidelines for the Brazilian Population*. The guidelines classify foods into categories according to the level of processing used in their production (fresh or minimally processed foods, processed foods, and ultra-processed foods) and recommend that the former should be the basis of Brazilians' nutrition. It may also be used to guide patients with chronic kidney disease, particularly those who require phosphatemia control. The guide also addresses economic, social, behavioral and cultural aspects that might be useful in guiding patients with hyperphosphatemia.^28^

### Phosphate binders

Considering the limitations associated with P restriction and P removal by dialysis, P binders are necessary for almost all patients undergoing dialysis. In theory, P binders should prevent or treat hyperphosphatemia. However, in clinical practice it is observed that the effect of binders is limited. The main P binders used in our context, as well as their characteristics, are listed in Table 2.

**Table 2.** Main phosphate binders with their respective characteristics

Binder	Binder capacity	Advantages	Side effects
Calcium carbonate (40% elemental calcium)	Low	Low cost	- Constipation - Hypercalcemia and metastatic calcification
Calcium acetate (25% elemental calcium)	Moderate	Higher binder capacity with lower calcium supply than calcium carbonate	- Constipation and nausea - Hypercalcemia and metastatic calcification
Sevelamer hydrochloride	Moderate	Contains no aluminum or calcium	- Diarrhea or constipation, flatulence, nausea and dyspepsia

The choice of the type of binder and the dose to be prescribed will depend on some factors. First, at meals where the amount of P is higher, the binder should be prescribed in larger amounts, and at those meals where there is no P-rich food, there is no need for a binder. Snacks or foods with high amounts of P, ingested at any time, should always be associated with binders. There are no established doses for the prescription of binders based on the amount of P in the diet. Thus, since eating habits are dynamic and the consumption of phosphorus sources varies from day to day and from meal to meal, it is essential to guide the patient regarding the need to take the binder or not, depending on the consumption of a given meal, in order to improve the binding process of this mineral. Frequent follow-up is the best way to assess prescription adequacy, making adjustments when necessary. Binders should be taken with meals, in order to allow the best mixture with the food. It is important that the patient understands how binders act, so that the best adherence is obtained and, consequently, the best results.

 Another consideration is serum Ca levels. Patients with hypercalcemia or vascular calcification should not use Ca-containing binders, and for those with calcemia at the upper limit of normality, the prescribed dose of Ca-based binders should be very cautious. If this is the only option, use Ca acetate. In summary, in patients receiving P-lowering treatment, the dose of Ca-containing binders should be restricted^29^. 

If Ca-based binders are contraindicated, sevelamer hydrochloride should always be used. Attention should be drawn to those patients taking 1,25-hydroxyvitamin D (calcitriol), since this hormone promotes increased intestinal absorption of Ca and P. The observation and monitoring of PTH levels throughout the treatment are also necessary, since SHP often proves to be resistant to clinical treatment, a situation that makes it impossible to decrease serum P, even with dietary restriction and massive use of binders. Furthermore, in the opposite situation, i. e., in relative hypoparathyroidism, when bone turnover is decreased, the reduced incorporation of P by the bone causes hyperphosphatemia to be maintained. In both cases, other treatment options should be considered, and it is important that the patient be informed about the reasons for treatment failure. The assessment of the proposed treatment should happen periodically, so that dietary and drug adjustments may be applied.

Finally, the success of the therapy fundamentally depends on the patient's participation. Thus, the guidelines should be clear and objective and the entire multidisciplinary team should be involved, especially the nutritionist. When dietary control and the use of P binders are insufficient, changes in dialysis prescription could be an adjunctive measure. Conventional dialysis treatment is insufficient to maintain a negative P balance in most dialysis patients. This is obvious when we compare the P clearance capacity of a 4-hour HD session, which is approximately 900 mg of P^30^, with the daily ingested amount, which is up to 1,000 mg/day, in a recommended diet with 1.0 to 1.2 g protein/kg/day^27^. Even changes in dialysate composition and flow, as well as in the type of capillary membrane, have not been shown to be effective in improving P clearance^31^ ^,^ ^32^. Peritoneal dialysis (PD), on the other hand, may provide a P control that is slightly better than HD, but still insufficient^33^. 

Inadequate removal of P by conventional HD is due to its own kinetics. P is a predominantly intracellular element. During the first hour of an HD session, there is a rapid removal of P, which peaks around 120 minutes. Thereafter, the removal rate drops and remains around half that of the initial phase, but without any change in serum P. Finally, there may be a post-dialytic rebound in which P levels may even exceed those at the beginning of the dialysis session^30^ ^,^ ^34^. Therefore, the kinetics of P removal follows a two-phase model. Initially, removal of P from the extracellular compartment occurs, followed by a flow of P from the intra to the extracellular compartment, which maintains its serum level constant throughout the remainder of the treatment. It is precisely the rate of P efflux into the dialysate during the first hours of dialysis and the rate of mobilization between the intra- and extracellular compartments that limit P removal. Hence, the frequency and duration of dialysis sessions directly correlate with adequate phosphatemia control.

The effects of new HD patterns, such as daily, prolonged nocturnal, and hemodiafiltration, on P control have been studied^35^ ^-^ ^39^. A universal finding of these studies is improved P control, with reduced or even discontinued use of P binders. In addition, better control of PTH and the Ca x P product is obtained^38^. Although promising, not all of these dialysis modalities are part of our daily practice. In cases of severe hyperphosphatemia, we could always resort to an increased number of weekly dialysis sessions or their duration, although sometimes there is resistance from the patient, due to the direct interference in his daily life. Furthermore, since conventional HD is a limited method for P control, patient attendance and maintenance of dialysis adequacy are of paramount importance, avoiding the reduction of treatment time, a practice that has become frequent in our setting.
